# (*Z*)-3-(1-Chloro­prop-1-en­yl)-2-methyl-1-phenyl­sulfonyl-1*H*-indole

**DOI:** 10.1107/S1600536813030730

**Published:** 2013-11-16

**Authors:** M. Umadevi, V. Saravanan, R. Yamuna, A. K. Mohanakrishnan, G. Chakkaravarthi

**Affiliations:** a Research Scholar (Chemistry), Bharathiyar University, Coimbatore 641 046, Tamilnadu, India; b Department of Organic Chemistry, University of Madras, Guindy Campus, Chennai 600 025, India; c Department of Sciences, Chemistry and Materials Research Lab, Amrita Vishwa Vidyapeetham University, Ettimadai, Coimbatore 641 112, India; d Department of Physics, CPCL Polytechnic College, Chennai 600 068, India

## Abstract

In the title compound, C_18_H_16_ClNO_2_S, the indole ring system forms a dihedral angle of 75.07 (8)° with the phenyl ring. The mol­ecular structure is stabilized by a weak intra­molecular C—H⋯O hydrogen bond. In the crystal, mol­ecules are linked by weak C—H⋯O hydrogen bonds, forming a chain along [10-1]. C—H⋯π inter­actions are also observed, leading to a three-dimensional network.

## Related literature

For the biological activity of indole derivatives, see: Okabe & Adachi (1998 ▶); Schollmeyer *et al.* (1995 ▶). For related structures, see: Chakkaravarthi *et al.* (2007 ▶, 2008 ▶).
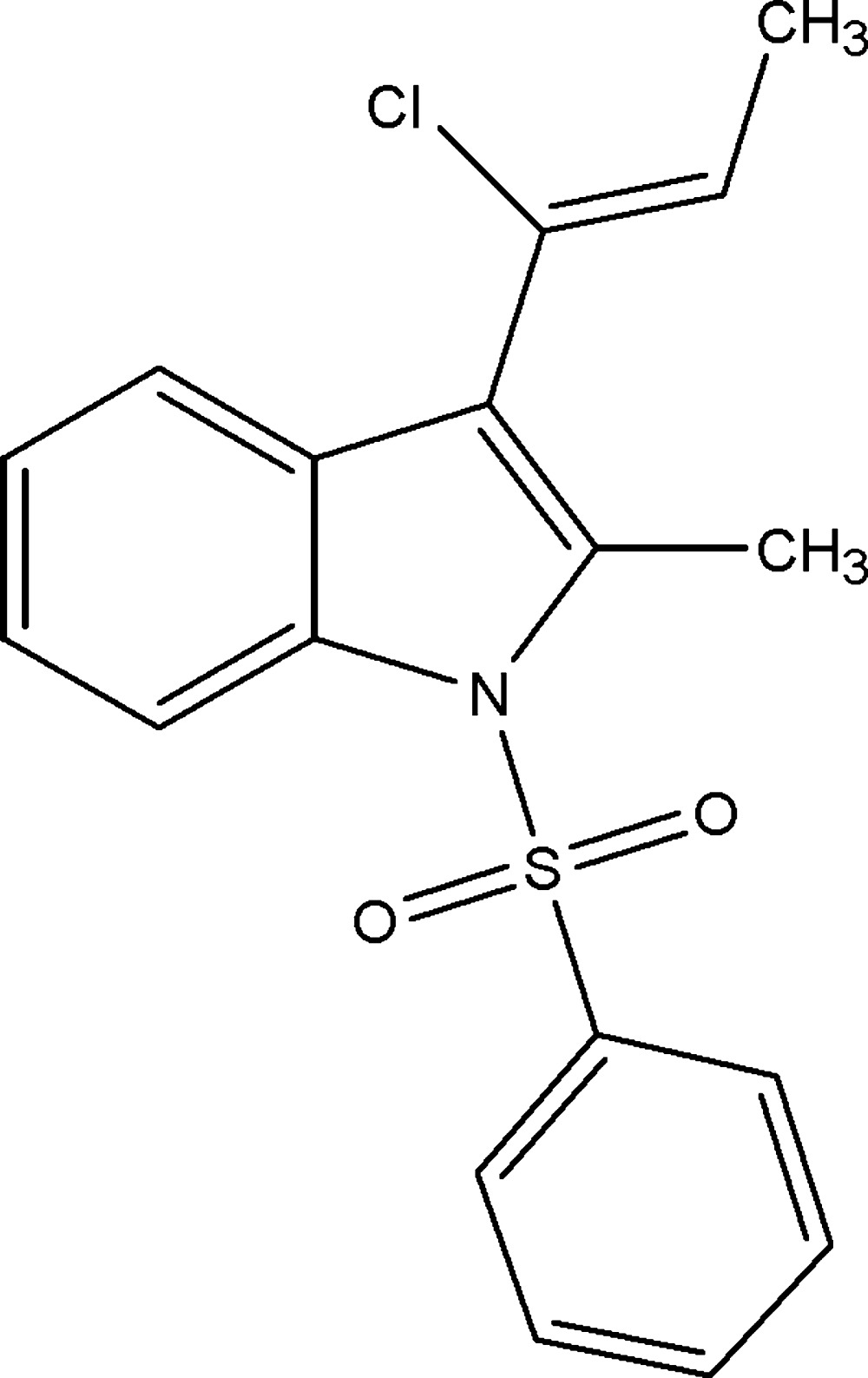


## Experimental

### 

#### Crystal data

C_18_H_16_ClNO_2_S
*M* *_r_* = 345.83
Monoclinic, 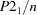

*a* = 12.5204 (10) Å
*b* = 10.4962 (7) Å
*c* = 12.983 (1) Å
β = 98.892 (2)°
*V* = 1685.7 (2) Å^3^
*Z* = 4
Mo *K*α radiation
μ = 0.36 mm^−1^
*T* = 295 K
0.28 × 0.24 × 0.18 mm

#### Data collection

Bruker Kappa APEXII diffractometer
Absorption correction: multi-scan (*SADABS*; Sheldrick, 1996 ▶) *T*_min_ = 0.906, *T*_max_ = 0.938
20036 measured reflections
4705 independent reflections
3357 reflections with *I* > 2σ(*I*)
*R*_int_ = 0.032

#### Refinement

*R*[*F* ^2^ > 2σ(*F* ^2^)] = 0.042
*wR*(*F* ^2^) = 0.118
*S* = 1.03
4705 reflections
210 parameters
H-atom parameters constrained
Δρ_max_ = 0.26 e Å^−3^
Δρ_min_ = −0.38 e Å^−3^

### 

Data collection: *APEX2* (Bruker, 2004 ▶); cell refinement: *SAINT* (Bruker, 2004 ▶); data reduction: *SAINT*; program(s) used to solve structure: *SHELXS97* (Sheldrick, 2008 ▶); program(s) used to refine structure: *SHELXL97* (Sheldrick, 2008 ▶); molecular graphics: *PLATON* (Spek, 2009 ▶); software used to prepare material for publication: *SHELXL97*.

## Supplementary Material

Crystal structure: contains datablock(s) I, global. DOI: 10.1107/S1600536813030730/is5320sup1.cif

Structure factors: contains datablock(s) I. DOI: 10.1107/S1600536813030730/is5320Isup2.hkl

Supplementary material file. DOI: 10.1107/S1600536813030730/is5320Isup3.cml

Additional supplementary materials:  crystallographic information; 3D view; checkCIF report

## Figures and Tables

**Table 1:** Hydrogen-bond geometry (Å, °) *Cg*1 is the centroid of the N1/C7/C12–C14 ring.

*D*—H⋯*A*	*D*—H	H⋯*A*	*D*⋯*A*	*D*—H⋯*A*
C8—H8⋯O1	0.93	2.39	2.973 (2)	120
C10—H10⋯O2^i^	0.93	2.49	3.364 (2)	157
C5—H5⋯*Cg*1^ii^	0.93	2.82	3.477 (2)	128

Symmetry codes: (i) 
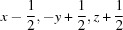
; (ii) 
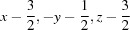
.

**Fig. 1.**
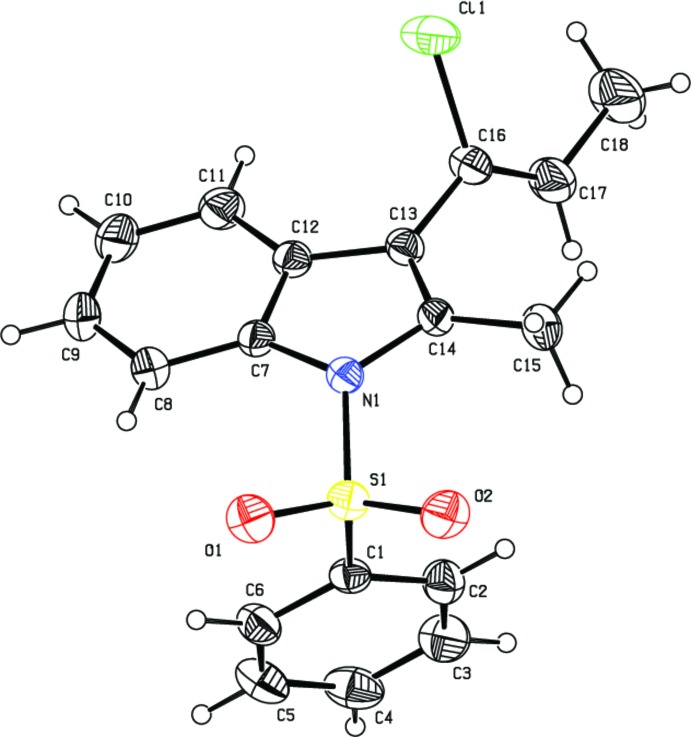
The molecular structure of the title compound, with atom labels and 30% probability displacement ellipsoids for non-H atoms.

**Fig. 2.**
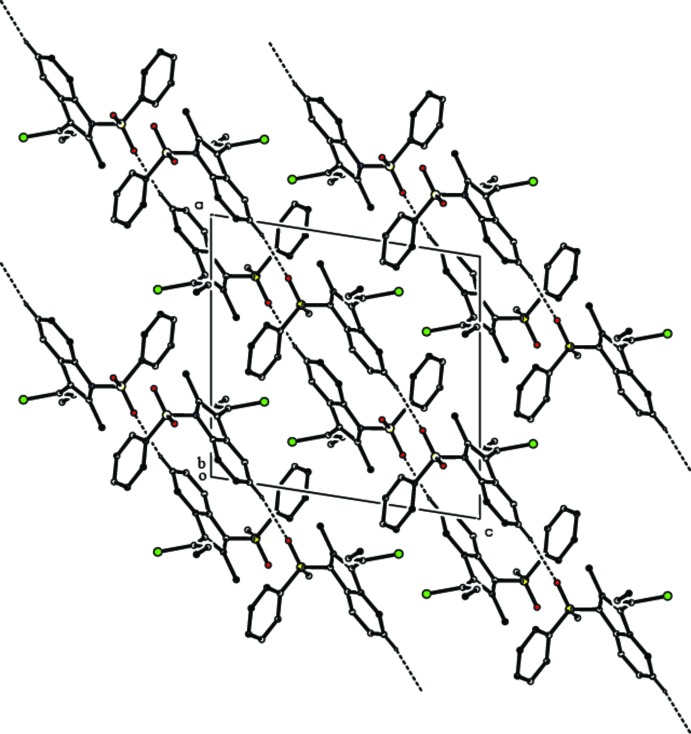
A packing diagram of the title compound, view down the b axis. Intermolecular hydrogen bonds are shown as dashed lines. H atoms not involving hydrogen bonding have been omitted.

## supplementary crystallographic information

### 1. Comment

The indole derivatives are known to exhibit anti-bacterial and anti-tumour activities (Okabe & Adachi, 1998; Schollmeyer *et al.*, 1995). We herein report the crystal structure of the title compound (I), (Fig. 1). The geometric parameters of (I) are comparable with the reported similar structures (Chakkaravarthi *et al.*, 2007, 2008). The phenyl ring forms a dihedral angle of 75.07 (8)° with the indole ring system. The five-membered (N1/C7/C12–C14) and six-membered (C7–C12) rings in the indole ring system are planar, with a dihedral angle of 0.38 (9)° between these rings. The bond angles around N1 (351.5°) indicate the *sp*^2^ hybridization of N1 atom. The molecular structure is stabilized by a weak intramolecular C—H···O (Table 1) hydrogen bond. The crystal structure exhibits weak intermolecular C—H···O (Fig. 2) and C—H···π (Table 1) interactions.

### 2. Experimental

To a solution of 2-methyl-1-(phenylsulfonyl)-1*H*-indole (1 g, 3.69 mmol) in dry dichloromethane (20 ml), AlCl_3_ (1.47 g, 11.07 mmol) and propionic anhydride (0.71 ml, 5.53 mmol) were added at 0 °C and stirred for 3 h. Then, the reaction mixture was washed with saturated NaHCO_3_ (2 × 10 ml) solution, followed by water (3 × 10 ml) and dried (Na_2_SO_4_). Removal of the solvent followed a column chromatographic purification (Silica gel; hexane-ethyl acetate, 95:5) afforded the title compound, suitable for X-Ray diffraction quality crystals.

### 3. Refinement

H atoms were positioned geometrically and refined using riding model, with C—H = 0.93 Å and *U*_iso_(H) = 1.2*U*_eq_(C) for CH, and C—H = 0.96 Å and *U*_iso_(H) = 1.5*U*_eq_(C) for CH_3_.

### Crystal data

**Table:**

C_18_H_16_ClNO_2_S	*F*(000) = 720
*M**_r_* = 345.83	*D*_x_ = 1.363 Mg m^−^^3^
Monoclinic, *P*2_1_/*n*	Mo *K*α radiation, λ = 0.71073 Å
Hall symbol: -P 2yn	Cell parameters from 6694 reflections
*a* = 12.5204 (10) Å	θ = 2.1–27.9°
*b* = 10.4962 (7) Å	µ = 0.36 mm^−^^1^
*c* = 12.983 (1) Å	*T* = 295 K
β = 98.892 (2)°	Block, colourless
*V* = 1685.7 (2) Å^3^	0.28 × 0.24 × 0.18 mm
*Z* = 4	

### Data collection

**Table:**

Bruker Kappa APEXII diffractometer	4705 independent reflections
Radiation source: fine-focus sealed tube	3357 reflections with *I* > 2σ(*I*)
Graphite monochromator	*R*_int_ = 0.032
ω and φ scan	θ_max_ = 29.6°, θ_min_ = 2.1°
Absorption correction: multi-scan (*SADABS*; Sheldrick, 1996)	*h* = −17→17
*T*_min_ = 0.906, *T*_max_ = 0.938	*k* = −14→8
20036 measured reflections	*l* = −17→17

### Refinement

**Table:**

Refinement on *F*^2^	Primary atom site location: structure-invariant direct methods
Least-squares matrix: full	Secondary atom site location: difference Fourier map
*R*[*F*^2^ > 2σ(*F*^2^)] = 0.042	Hydrogen site location: inferred from neighbouring sites
*wR*(*F*^2^) = 0.118	H-atom parameters constrained
*S* = 1.03	*w* = 1/[σ^2^(*F*_o_^2^) + (0.048*P*)^2^ + 0.5443*P*] where *P* = (*F*_o_^2^ + 2*F*_c_^2^)/3
4705 reflections	(Δ/σ)_max_ < 0.001
210 parameters	Δρ_max_ = 0.26 e Å^−^^3^
0 restraints	Δρ_min_ = −0.38 e Å^−^^3^

### Special details

**Table:**

Refinement. Refinement of *F*^2^ against ALL reflections. The weighted *R*-factor *wR* and goodness of fit *S* are based on *F*^2^, conventional *R*-factors *R* are based on *F*, with *F* set to zero for negative *F*^2^. The threshold expression of *F*^2^ > σ(*F*^2^) is used only for calculating *R*-factors(gt) *etc*. and is not relevant to the choice of reflections for refinement. *R*-factors based on *F*^2^ are statistically about twice as large as those based on *F*, and *R*- factors based on ALL data will be even larger.

### Fractional atomic coordinates and isotropic or equivalent isotropic displacement parameters (Å^2^)

**Table:**

	*x*	*y*	*z*	*U*_iso_*/*U*_eq_	
C1	0.59725 (13)	0.25271 (15)	0.25702 (12)	0.0409 (4)	
C2	0.61126 (16)	0.36677 (19)	0.20771 (15)	0.0538 (4)	
H2	0.6791	0.4046	0.2140	0.065*	
C3	0.52304 (19)	0.4234 (2)	0.14909 (17)	0.0662 (6)	
H3	0.5310	0.5006	0.1159	0.079*	
C4	0.42317 (19)	0.3664 (2)	0.13940 (17)	0.0677 (6)	
H4	0.3641	0.4048	0.0990	0.081*	
C5	0.41016 (17)	0.2544 (2)	0.18844 (18)	0.0658 (6)	
H5	0.3421	0.2170	0.1817	0.079*	
C6	0.49712 (15)	0.19547 (18)	0.24823 (16)	0.0532 (4)	
H6	0.4883	0.1188	0.2818	0.064*	
C7	0.64323 (12)	0.27161 (16)	0.50763 (12)	0.0387 (3)	
C8	0.56922 (15)	0.17868 (18)	0.52523 (15)	0.0506 (4)	
H8	0.5671	0.0997	0.4925	0.061*	
C9	0.49922 (17)	0.2087 (2)	0.59334 (17)	0.0616 (5)	
H9	0.4485	0.1487	0.6068	0.074*	
C10	0.50223 (17)	0.3261 (2)	0.64240 (17)	0.0643 (6)	
H10	0.4534	0.3435	0.6877	0.077*	
C11	0.57600 (16)	0.4172 (2)	0.62521 (15)	0.0548 (5)	
H11	0.5778	0.4958	0.6585	0.066*	
C12	0.64785 (13)	0.38933 (16)	0.55705 (13)	0.0402 (4)	
C13	0.73423 (13)	0.46134 (16)	0.52297 (13)	0.0404 (4)	
C14	0.77940 (13)	0.39021 (16)	0.45452 (13)	0.0403 (4)	
C15	0.87719 (15)	0.4191 (2)	0.40629 (17)	0.0599 (5)	
H15A	0.9096	0.4967	0.4352	0.090*	
H15B	0.8568	0.4287	0.3323	0.090*	
H15C	0.9282	0.3506	0.4203	0.090*	
C16	0.77116 (14)	0.58673 (17)	0.56392 (14)	0.0474 (4)	
C17	0.7704 (2)	0.6928 (2)	0.51122 (18)	0.0678 (6)	
H17	0.7463	0.6881	0.4399	0.081*	
C18	0.8047 (3)	0.8218 (2)	0.5545 (2)	0.0989 (10)	
H18A	0.7573	0.8856	0.5198	0.148*	
H18B	0.8775	0.8386	0.5434	0.148*	
H18C	0.8014	0.8234	0.6278	0.148*	
N1	0.72580 (10)	0.26962 (13)	0.44364 (10)	0.0389 (3)	
O1	0.68061 (11)	0.05793 (12)	0.36391 (11)	0.0580 (3)	
O2	0.80267 (11)	0.20029 (14)	0.28742 (11)	0.0606 (4)	
S1	0.70889 (3)	0.18303 (4)	0.33504 (3)	0.04316 (13)	
Cl1	0.81805 (5)	0.58714 (5)	0.69822 (4)	0.07334 (19)	

### Atomic displacement parameters (Å^2^)

**Table:**

	*U*^11^	*U*^22^	*U*^33^	*U*^12^	*U*^13^	*U*^23^
C1	0.0475 (9)	0.0363 (9)	0.0386 (8)	0.0013 (7)	0.0059 (7)	−0.0079 (6)
C2	0.0581 (11)	0.0478 (11)	0.0547 (11)	−0.0050 (8)	0.0061 (9)	0.0042 (8)
C3	0.0830 (16)	0.0484 (12)	0.0628 (13)	0.0038 (10)	−0.0028 (11)	0.0088 (9)
C4	0.0729 (14)	0.0563 (13)	0.0647 (13)	0.0113 (11)	−0.0182 (11)	−0.0081 (10)
C5	0.0532 (12)	0.0609 (14)	0.0768 (15)	−0.0059 (9)	−0.0107 (10)	−0.0115 (11)
C6	0.0562 (11)	0.0432 (10)	0.0575 (11)	−0.0082 (8)	0.0008 (9)	−0.0047 (8)
C7	0.0361 (8)	0.0442 (9)	0.0353 (8)	−0.0018 (6)	0.0041 (6)	0.0017 (6)
C8	0.0532 (10)	0.0487 (11)	0.0502 (10)	−0.0107 (8)	0.0086 (8)	0.0016 (8)
C9	0.0551 (11)	0.0723 (14)	0.0602 (12)	−0.0187 (10)	0.0178 (9)	0.0050 (10)
C10	0.0534 (11)	0.0863 (16)	0.0586 (12)	−0.0065 (10)	0.0260 (10)	−0.0055 (11)
C11	0.0534 (11)	0.0625 (12)	0.0507 (11)	−0.0006 (9)	0.0147 (8)	−0.0114 (9)
C12	0.0381 (8)	0.0443 (9)	0.0372 (8)	−0.0003 (6)	0.0030 (6)	0.0004 (6)
C13	0.0393 (8)	0.0400 (9)	0.0403 (8)	−0.0028 (6)	0.0009 (6)	0.0010 (6)
C14	0.0367 (8)	0.0443 (9)	0.0392 (8)	−0.0053 (6)	0.0032 (6)	0.0018 (6)
C15	0.0452 (10)	0.0750 (14)	0.0617 (12)	−0.0163 (9)	0.0151 (9)	−0.0058 (10)
C16	0.0464 (9)	0.0449 (10)	0.0484 (10)	−0.0041 (7)	−0.0008 (7)	−0.0033 (7)
C17	0.0869 (16)	0.0502 (13)	0.0620 (13)	−0.0157 (10)	−0.0022 (11)	0.0037 (9)
C18	0.147 (3)	0.0493 (15)	0.094 (2)	−0.0298 (15)	−0.0015 (18)	0.0022 (12)
N1	0.0374 (7)	0.0413 (8)	0.0381 (7)	−0.0024 (5)	0.0059 (5)	−0.0021 (5)
O1	0.0720 (9)	0.0340 (7)	0.0679 (9)	0.0075 (6)	0.0105 (7)	−0.0009 (6)
O2	0.0519 (8)	0.0711 (10)	0.0631 (9)	0.0087 (6)	0.0228 (6)	−0.0116 (7)
S1	0.0456 (2)	0.0383 (2)	0.0463 (2)	0.00578 (16)	0.00928 (17)	−0.00582 (17)
Cl1	0.1009 (4)	0.0574 (3)	0.0532 (3)	−0.0016 (3)	−0.0152 (3)	−0.0087 (2)

### Geometric parameters (Å, º)

**Table:**

C1—C6	1.379 (2)	C11—C12	1.387 (2)
C1—C2	1.382 (2)	C11—H11	0.9300
C1—S1	1.7541 (17)	C12—C13	1.444 (2)
C2—C3	1.376 (3)	C13—C14	1.350 (2)
C2—H2	0.9300	C13—C16	1.467 (2)
C3—C4	1.374 (3)	C14—N1	1.429 (2)
C3—H3	0.9300	C14—C15	1.490 (2)
C4—C5	1.359 (3)	C15—H15A	0.9600
C4—H4	0.9300	C15—H15B	0.9600
C5—C6	1.382 (3)	C15—H15C	0.9600
C5—H5	0.9300	C16—C17	1.306 (3)
C6—H6	0.9300	C16—Cl1	1.7517 (18)
C7—C8	1.388 (2)	C17—C18	1.502 (3)
C7—C12	1.389 (2)	C17—H17	0.9300
C7—N1	1.4229 (19)	C18—H18A	0.9600
C8—C9	1.374 (3)	C18—H18B	0.9600
C8—H8	0.9300	C18—H18C	0.9600
C9—C10	1.385 (3)	N1—S1	1.6634 (14)
C9—H9	0.9300	O1—S1	1.4251 (14)
C10—C11	1.371 (3)	O2—S1	1.4207 (13)
C10—H10	0.9300		
			
C6—C1—C2	121.21 (17)	C7—C12—C13	107.68 (14)
C6—C1—S1	119.95 (14)	C14—C13—C12	108.79 (15)
C2—C1—S1	118.82 (14)	C14—C13—C16	126.44 (15)
C3—C2—C1	118.84 (19)	C12—C13—C16	124.58 (15)
C3—C2—H2	120.6	C13—C14—N1	108.47 (14)
C1—C2—H2	120.6	C13—C14—C15	128.24 (16)
C4—C3—C2	120.3 (2)	N1—C14—C15	122.86 (15)
C4—C3—H3	119.9	C14—C15—H15A	109.5
C2—C3—H3	119.9	C14—C15—H15B	109.5
C5—C4—C3	120.5 (2)	H15A—C15—H15B	109.5
C5—C4—H4	119.8	C14—C15—H15C	109.5
C3—C4—H4	119.8	H15A—C15—H15C	109.5
C4—C5—C6	120.6 (2)	H15B—C15—H15C	109.5
C4—C5—H5	119.7	C17—C16—C13	126.77 (18)
C6—C5—H5	119.7	C17—C16—Cl1	119.57 (15)
C1—C6—C5	118.62 (19)	C13—C16—Cl1	113.66 (13)
C1—C6—H6	120.7	C16—C17—C18	126.4 (2)
C5—C6—H6	120.7	C16—C17—H17	116.8
C8—C7—C12	122.00 (15)	C18—C17—H17	116.8
C8—C7—N1	130.50 (16)	C17—C18—H18A	109.5
C12—C7—N1	107.49 (13)	C17—C18—H18B	109.5
C9—C8—C7	116.92 (18)	H18A—C18—H18B	109.5
C9—C8—H8	121.5	C17—C18—H18C	109.5
C7—C8—H8	121.5	H18A—C18—H18C	109.5
C8—C9—C10	121.70 (18)	H18B—C18—H18C	109.5
C8—C9—H9	119.2	C7—N1—C14	107.56 (13)
C10—C9—H9	119.2	C7—N1—S1	119.61 (11)
C11—C10—C9	121.08 (18)	C14—N1—S1	124.33 (11)
C11—C10—H10	119.5	O2—S1—O1	119.18 (8)
C9—C10—H10	119.5	O2—S1—N1	107.08 (8)
C10—C11—C12	118.48 (18)	O1—S1—N1	106.54 (8)
C10—C11—H11	120.8	O2—S1—C1	109.51 (8)
C12—C11—H11	120.8	O1—S1—C1	109.14 (8)
C11—C12—C7	119.81 (16)	N1—S1—C1	104.32 (7)
C11—C12—C13	132.51 (17)		
			
C6—C1—C2—C3	0.1 (3)	C14—C13—C16—C17	66.3 (3)
S1—C1—C2—C3	−178.03 (16)	C12—C13—C16—C17	−119.2 (2)
C1—C2—C3—C4	−0.5 (3)	C14—C13—C16—Cl1	−114.73 (18)
C2—C3—C4—C5	0.7 (4)	C12—C13—C16—Cl1	59.8 (2)
C3—C4—C5—C6	−0.4 (3)	C13—C16—C17—C18	177.7 (2)
C2—C1—C6—C5	0.2 (3)	Cl1—C16—C17—C18	−1.3 (4)
S1—C1—C6—C5	178.30 (15)	C8—C7—N1—C14	−179.54 (17)
C4—C5—C6—C1	0.0 (3)	C12—C7—N1—C14	−0.88 (17)
C12—C7—C8—C9	0.7 (3)	C8—C7—N1—S1	31.1 (2)
N1—C7—C8—C9	179.20 (18)	C12—C7—N1—S1	−150.22 (12)
C7—C8—C9—C10	−0.1 (3)	C13—C14—N1—C7	1.30 (18)
C8—C9—C10—C11	−0.4 (4)	C15—C14—N1—C7	174.34 (16)
C9—C10—C11—C12	0.2 (3)	C13—C14—N1—S1	148.83 (12)
C10—C11—C12—C7	0.4 (3)	C15—C14—N1—S1	−38.1 (2)
C10—C11—C12—C13	−179.39 (19)	C7—N1—S1—O2	179.44 (12)
C8—C7—C12—C11	−0.9 (3)	C14—N1—S1—O2	35.51 (15)
N1—C7—C12—C11	−179.69 (16)	C7—N1—S1—O1	−51.99 (14)
C8—C7—C12—C13	178.96 (16)	C14—N1—S1—O1	164.08 (13)
N1—C7—C12—C13	0.17 (18)	C7—N1—S1—C1	63.40 (14)
C11—C12—C13—C14	−179.51 (19)	C14—N1—S1—C1	−80.53 (14)
C7—C12—C13—C14	0.65 (19)	C6—C1—S1—O2	143.30 (15)
C11—C12—C13—C16	5.2 (3)	C2—C1—S1—O2	−38.55 (16)
C7—C12—C13—C16	−174.67 (15)	C6—C1—S1—O1	11.19 (17)
C12—C13—C14—N1	−1.20 (18)	C2—C1—S1—O1	−170.66 (13)
C16—C13—C14—N1	174.01 (15)	C6—C1—S1—N1	−102.37 (15)
C12—C13—C14—C15	−173.75 (18)	C2—C1—S1—N1	75.78 (15)
C16—C13—C14—C15	1.5 (3)		

### Hydrogen-bond geometry (Å, º)

*Cg*1 is the centroid of the N1/C7/C12–C14 ring.

**Table:**

*D*—H···*A*	*D*—H	H···*A*	*D*···*A*	*D*—H···*A*
C8—H8···O1	0.93	2.39	2.973 (2)	120
C10—H10···O2^i^	0.93	2.49	3.364 (2)	157
C5—H5···*Cg*1^ii^	0.93	2.82	3.477 (2)	128

Symmetry codes: (i) *x*−1/2, −*y*+1/2, *z*+1/2; (ii) *x*−3/2, −*y*−1/2, *z*−3/2.
